# Integrative physiological, biochemical, and metabolomic analyses reveal complex drought and zinc stress tolerance in a novel *Miscanthus* hybrid

**DOI:** 10.3389/fpls.2025.1556144

**Published:** 2025-10-29

**Authors:** Monirul Islam, Amjad Ali, Leilei Zhang, Andrea Ferrarini, Luigi Lucini, John Clifton-Brown, Stefano Amaducci

**Affiliations:** ^1^ Department of Sustainable Crop Production, Università Cattolica del Sacro Cuore, Piacenza, PC, Italy; ^2^ Department of Biochemistry and Molecular Biology, University of Nevada, Reno, NV, United States; ^3^ Department of Sustainable Food Process, Università Cattolica del Sacro Cuore, Piacenza, PC, Italy; ^4^ CRAST Research Centre, Università Cattolica del Sacro Cuore, Piacenza, PC, Italy; ^5^ Justus Liebig University, Gießen, Germany

**Keywords:** *Miscanthus*, gas exchange, untargeted metabolomics, antioxidant enzymes, drought stress, zinc stress

## Abstract

Stresses caused by drought and heavy metals (HMs) adversely affect the establishment and yield potential of *Miscanthus* plants. These stresses are particularly acute on lower quality ‘contaminated and marginal-lands’ less suitable for food production. In our prior research assessing drought and zinc stress tolerance across seven novel *Miscanthus* hybrids, a *M. sacchariflorus* × *M. sinensis* hybrid ‘GRC10’ exhibited superior stress tolerance and biomass production. This study investigated the effects of drought (D), zinc (Zn) stress, and their combination (D + Zn) on stress tolerance in the *Miscanthus* GRC10 using untargeted metabolomics to uncover stress tolerance mechanisms. Synchronous measurements of growth parameters, leaf gas exchange parameters, the maximum quantum yield of photosystem II (Fv/Fm), performance index (PI-ABS), antioxidant enzyme activity, proline, and malondialdehyde (MDA) production were made to elucidate associations. Both D, Zn, and combination (D + Zn) stress induced a broad metabolic reprogramming of secondary metabolism and hormone synthesis pathways. Fatty acid derivatives, nitrogen-containing compounds, hormone/signal-related compounds (jasmonate), and secondary metabolites (phenylpropanoids, N-containing compounds, and terpenes) showed significant (*p* < 0.05) abundance changes in response to D, Zn, and its combination D + Zn stress. Drought, Zn, and combination D + Zn stress treatments increased proline accumulation (*p* < 0.0001), antioxidant enzyme activities (*p* < 0.05), including superoxide dismutase (SOD), ascorbate peroxidase (APX), glutathione reductase (GR), and decreased levels of MDA. Overall, these responses indicate that the *Miscanthus* GRC10 hybrid displays a complex response to drought and Zn stresses that confers growth resilience in Zn-contaminated and drought-prone lands.

## 1 Introduction

The extent of arid land and areas contaminated with heavy metals (HMs) is expanding, resulting in significant consequences for future crop productivity. The environmental stresses resulting from climate change and human activities such as industrial activities, mining, fossil fuels, waste disposal, agriculture, and land use changes are anticipated to affect crop productivity and reduce the area of available land for agriculture by 2−9% globally and by 11−17% within Europe (Zhang and Cai, 2011). Marginal lands, unsuitable for cultivating edible crops, might be better used for the cultivation of biomass crops (Mehmood et al., 2017). For this purpose, perennial biomass crops, which are resilient to environmental stress, can help reduce greenhouse gas emissions while producing bioenergy and renewable products without competing with food crops (Popp et al., 2014; Amaducci et al., 2017).

In nature, crops are often exposed to combinations of stresses, such as salinity, heat, drought, flooding, and HMs toxicity during the growing season (Feizabadi et al., 2021). However, the interaction between drought and HMs remains largely unexplored. Drought stress reduces photosynthetic activity, alters cell-wall elasticity, and induces toxic metabolite production, leading to cell death, whereas heavy metals disrupt photosynthesis, damage cellular structures, affect water uptake and transport, and influence stomatal regulation in plants (Soba et al., 2022; Michaletti et al., 2018). Excessive soil heavy metal content, including zinc (Zn), disrupts chlorophyll synthesis and reduces photosynthesis rate, transpiration rate, and stomatal conductance in C_3_ and C_4_ photosynthesis bioenergy crops (Adamczyk-Szabela et al., 2023; Andrejić et al., 2018). Plants sense and react rapidly to slight changes in water status or heavy metal toxicity through physiological, cellular, and molecular mechanisms. However, such responses are determined by the intensity, duration, and rate of progression of the drought and HMs stress exposure (Lajayer et al., 2017).

Plants combat oxidative stress primarily through intrinsic redox regulation, employing enzymatic components like superoxide dismutase (SOD), peroxidase (POD), glutathione reductase (GR), catalase (CAT), and ascorbate peroxidase (APX), and non-enzymatic compounds such as phenolic acids, alkaloids, flavonoids, and carotenoids (Mishra et al., 2023). Alternatively, plant response to drought and heavy metal stress is influenced significantly by plant hormones like abscisic acid (ABA) and jasmonic acid (JA) (Zhang et al., 2006). Elevated JA levels enhance ABA-mediated tolerance to drought and HMs stress, and these ABA and JA levels commonly serve as reliable indicators for plant stress tolerance (de Ollas et al., 2013; Alam et al., 2014). Moreover, plants produce a diverse range of metabolites, such as amino acids, sugars, sugar alcohols, polyamines (PAs), and glycine betaine (GB) (Parida et al., 2008; Medeiros et al., 2012). Drought-tolerant plants have demonstrated heightened turnover of carbohydrates as well (Des Marais et al., 2012). Proline, sugars, and glycine betaine are osmotically neutral metabolites that play important roles in osmotic adjustment under drought and HMs stress (Patel and Vora, 1985). PAs are nitrogen-containing polycationic compounds found universally in eukaryotic cells, with putrescine, spermidine, and spermine being most prominent (Montesinos-Pereira et al., 2014). Elevated PAs concentrations are strongly correlated with increased drought and HMs stress tolerance (Yang et al., 2007; Liang et al., 2013). As a result, analyzing the metabolic profiles of plants during oxidative stress is a valuable approach to evaluate their resilience and stress tolerance.

*Miscanthus* is a perennial bioenergy crop that achieves high biomass production on marginal lands due to high water-use efficiency and adaptability to harsh conditions (Amaducci et al., 2017). However, *Miscanthus* biomass production can be reduced by limited water availability and heavy metal soil contamination (Stone et al., 2010). To date, the physiological, biochemical, and metabolomics traits associated with drought and Zn stress tolerance in recently developed *Miscanthus* hybrids are poorly understood (Impollonia et al., 2022). Evaluation of the drought and Zn stress response of seven hybrids of *M. sinensis* × *M. sinensis*, *Miscanthus* × *giganteus*, and *M. sacchariflorus* × *M. sinensis* showed that *M. sacchariflorus* × *M. sinensis* GRC10 was the most resilient hybrid to both drought and Zn stress compared to the other hybrids (Islam et al., 2023). This study aims to investigate how *M. sinensis* × *M. sacchariflorus* GRC10 hybrid responds to drought, Zn stress, both individually and in combination, using comparative physiological, biochemical, and metabolomic analyses. Profiling secondary metabolites and key stress-response pathways, we seek to uncover the underlying mechanisms that govern *M. sinensis* × *M. sacchariflorus* GRC10 hybrid adaptation to drought, Zn, and combined stress.

## 2 Materials and methods

### 2.1 Plant material, growth conditions, and stress treatments

Seedlings of wild-sourced seed collected in China in 2008 by an international team led by John Clifton-Brown (who led the Aberystwyth (UK) breeding programme from 2004 to 2021) were raised in Canada and then imported into the US locations for field evaluation. In 2011, phenotypic selections made in Indiana’s continental climate were made by CERES’ breeder, Dr. Charlie Rodgers. Genotypes from both species were cloned to exploit inherent self-incompatibility and planted at a field site near College Station in Texas in isolated crossing blocks. In the crossing blocks, the parental clones were planted into alternate ‘A and B parental rows’ to generate bi-parental interspecific seeds. The warm climatic conditions in autumn induced synchronous flowering in both *M. sacchariflorus* and *M. sinensis* genotypes. In 2012/3 large quantities of interspecific seed were threshed separately from both parental genotypes. The seed was imported back into Europe for field trials, planted in 2013/4. A high yield potential for a hybrid coded GNT3 was recognized in field trials in both the UK (Lincoln) and in Germany (JKI, Braunschweig) in 2013-2016. Consequently, in 2017, GNT3 was selected for the GRACE project multi-location trials and recoded ‘GRC10’ for the GRACE project multilocation trials on marginal lands (Shepherd et al., 2023; Clifton-Brown et al., 2023).

Then the experiments were conducted in a control growth room of the Department of Sustainable Crop Production of the Università Cattolica del Sacro Cuore, Piacenza, Italy. The rhizomes of *M. sacchariflorus* x *M. sinensis* hybrid ‘GRC10’ were collected from a 4-year field trial at the Università Cattolica del Sacro Cuore, Piacenza (NW, Italy). After collecting, rhizomes were washed to remove soil, cut into 7–10 cm length (around 10 gm of FW) with several buds, and sowed 5 cm deep in 4 L round plastic pots with a commercial peat-humus, soil, and sand (3:1:1). Following two weeks of rhizome germination, uniform plants were transplanted into 16 L cylinder shaped pots filled with field soil and grown under PPFD of 800 µmol m^-2^s^-1^ light-emitting diodes (LED) with 16/8 h light/dark photoperiod. The temperature was set at a day/night cycle of 25/20°C.

To explore the effect of both drought and Zn stress on *M. sacchariflorus* × *M. sinensis* hybrid, four distinct treatments were applied: control (C) watering to maintain 80% field capacity (FC), drought (D) stress condition of 20% FC, Zn stress imposed by adding 400 mg ZnSO_4_ × 7H_2_O per kg of soil (dry mass, DM), and a combined treatment of drought at 20% FC and Zn stress of 400 mg ZnSO_4_.7H_2_O (D + Zn). For Zn stress, ZnSO_4_ was added to the soil before transferring the plants (2 weeks old) to 16 L pots, and drought stress was applied for one month. Plants were watered to 80% or 20% of field capacity every second day by weighing the pots and fertilized weekly with a modified half-strength of Hoagland’s solution (pH 6.0, EC 1.1 dS m^-1^). The experimental set-up was arranged in a completely randomized design (CRD) under factorial arrangement, and each treatment was replicated four times to ensure robust data analysis. After one month of treatment, all plants were tested for morphological and root biomass measurements and physiological analysis (leaf gas exchange and chlorophyll fluorescence), and after that, leaves were harvested in liquid N_2_ for further biochemical and metabolomics analysis. All the harvested samples were stored at -80°C until analysis.

### 2.2 Morphological and physiological traits

#### 2.2.1 Growth measurements and root biomass

All morphological measurements were evaluated for all treatments at the end of the experiment (30 days after treatment, DAT, and 60 days after germination). For the measurement of plant height, the length of the longest stem was measured from the base of the stem at soil level to the fully expanded ligule of the youngest leaf by using a graduated ruler. Similarly, the most expanded leaf length was measured using the graduated ruler. At the end of the experiment, after harvesting leaf samples, the roots of the identical plants were washed with tap water and dried at 70°C in an oven for 72 h. Subsequently, the dry weight of the roots was measured.

#### 2.2.2 Leaf gas exchange and chlorophyll fluorescence measurements

All parameters were measured using a portable infrared open gas exchange system with a 1.7-cm^2^ clamp-on leaf chamber (CIRAS-2, PP Systems International, Inc., Amesbury, MA, USA) (Tang et al., 2017). The measurements were performed for the parameters of the net photosynthetic rate (Pn), stomatal conductance (g_s_), and transpiration rate (E), at a flow rate of 2,000 µmol^-1^s^-1^ and saturated CO_2_ concentration of 400 µmol^-1^. Additionally, intrinsic (instantaneous) water use efficiency (iWUE) was calculated as Pn/gs at each measurement time. The conditions were the following in the leaf chamber: leaf temperature of 25°C, relative air humidity of 55%, and 400 µmol (CO_2_) mol^−1^. All the measurements were carried out with four technical replications on the second fully expanded leaf for all conditions (control and treatments) during the mid-morning period, and measurements were repeated on the last 3 days of the stress period.

Chlorophyll fluorescence measurements of photosystem II efficiency (PSII) and the maximum quantum yield (Fv/Fm) were performed on the same leaf of gas exchange with four technical replications for each plant after 30 days of stress. Measurements were executed with a pocket PEA (Plant efficiency analyzer) chlorophyll fluorimeter (Hansatech Instruments Ltd., King’s Lynn, UK), after at least one hour of dark-adaptation, and consisted of a light pulse of 3500 µmol m^-2^ s^-1^ provided by a single diode emitting at a peak wavelength of 627 nm. The initial and maximal fluorescence were determined to measure maximum PSII photochemical efficiency as Fv/Fm (ratio of variable fluorescence to maximum fluorescence), and the fast fluorescence transient for the determination of performance index (PI).

### 2.3 Biochemical assays

#### 2.3.1 Determination of leaf malondialdehyde and proline

For the determination of Malondialdehyde, 200 mg of leaf samples were centrifuged at 3,000 × *g* for 20 min after homogenization in 2 mL of 0.1% (w/v) trichloroacetic acid (TCA) as described previously by Ohkawa et al. (1979). Thereafter, 1 mL of the extract was mixed with 2 mL solution containing 0.5% (w/v) thiobarbituric (TBA) and 20% (w/v) TCA. The mixture was heated at 95°C for 30 min and promptly cooled in an ice bath for 20 min before centrifuging at 3, 000 × *g* for 5 min. The samples were aliquoted into two separate 2 ml tubes and centrifuged at 10,000 × *g* for 5 min. Lastly, the absorbance was measured at 532 and 600 nm after centrifugation. The solution containing 0.5% TBA and 20% TCA was used as a blank. The concentration of MDA was calculated using the formula: MDA (nmol g^−1^ FW) = [(OD532−OD600)] / (ϵ*FW), where FW is the fresh weight and ϵ the extinction coefficient (155 mM^−1^ cm^−1^). The values for MDA leaf content are expressed as μmol g^−1^ FW (fresh weight).

Proline content was estimated according to the method of Bates et al. (1973) and Verslues et al. (2007) with slight modifications. Briefly, leaf samples (200–300 mg) were ground in 5 mL of sulfosalicylic acid and then centrifuged at 10,000 × *g* for 10 min at 4°C. The collected supernatant was stored at -20°C in 1.5 mL Eppendorf tubes. For the assessment of proline level, 100 μL of leaf extraction was added with 1 mL of 1% ninhydrin solution, which contains a 60:40 ratio of glacial acetic acid: water, and boiled for 20 min in a water bath at 95°C. Then, the reaction was terminated by placing the samples on ice for 10 min. A 3 mL aliquot of toluene was added to the reaction mixture and vortexed for 20 sec, and the mixture was kept in the dark condition for 1h at room temperature. For the blank 100 μL of a 3% sulfosalicylic acid solution used instead of the plant extraction. The toluene phase was read at 520 nm with a microplate reader, and the concentration of proline was determined by using a standard curve of proline. Results were expressed in μmol g^−1^FW.

#### 2.3.2 Antioxidant enzyme assays: SOD, APX and GR

For enzyme extraction, 200 mg of frozen leaves were ground using a mortar and pestle in 4 ml of cold 50 mM K-phosphate buffer (pH 7.0), with 2 mM Na–ethylenediaminetetraacetic acid (EDTA) and 1% (w/v) polyvinyl–polyvinylpyrrolidone (PVPP). Homogenates were centrifuged at 10,000 × *g* for 10 minutes at 4°C, and supernatants were transferred to new tubes. The enzyme extracts were stored at -20°C before activity measurements, as described below.

For the determination of superoxide dismutase (SOD), a 1.5 mL mixture was added, containing 50 mM sodium carbonate/bicarbonate buffer (pH = 9.8), 0.1 mM EDTA, and 0.6 mM epinephrine (Goud and Kachole, 2012). Finally, the production of adrenochrome was measured in an ultraviolet-visible (UV-Vis) spectrophotometer at 475 nm after 4 min, and one unit of SOD activity is defined as the amount of enzyme required to prevent epinephrine oxidation by 50% under the experimental conditions.

The activity of ascorbate peroxidase (APX) was determined using the method of Halliwell and Foyer (1978), which involved mixing 100 μL of leaf extracts with 0.1 mM EDTA, 50 mM KP-buffer (pH 7.0), 0.1 mM H_2_O_2,_ and 0.5 mM ascorbic acid. Lastly, the absorbance was measured at 290 nm and calculated based on the extinction coefficient (2.8 mM-^1^ cm^−1^).

The quantification of glutathione reductase (GR) was carried out as described by Halliwell and Foyer (1978). In this procedure, 100 μL leaf extracts were mixed with 0.2 mol potassium phosphate (KP-buffer) (pH = 7.0), 1 mM Ethylenediaminetetraacetic acid (EDTA), 20 mM oxidized glutathione (GSSG), and 0.2 mM NADPH. Absorbance was measured at 340 nm, and the activity was calculated using the extinction coefficient (6.12 mM^−1^ cm^−1^).

### 2.4 Untargeted metabolomics analysis in leaves

Miscanthus, *M. sacchariflorus* × *M. sinensis* leaf tissues were prepared for untargeted metabolomics analysis by homogenizer-assisted solvent extraction as previously reported (Zhang et al., 2021). For this purpose, 1 g of frozen leaf tissues was extracted in a methanolic solution (80% methanol and 0.1% formic acid) (Merck KGaA, Darmstadt, Germany) by homogenization (Polytron^®^ PT 1200 E, Kinematica AG, Switzerland) for 10 min and subsequently centrifuged at 8,000 × *g* for 15 min at 4 °C. The supernatant was filtered with a 0.22 µm membrane and transferred to vials for analysis. The untargeted metabolomics analysis was conducted employing ultra-high performance liquid chromatography coupled with quadrupole time-of-flight mass spectrometry (UHPLC-QTOF/MS; Agilent Technologies, Santa Clara, CA, USA), following the methodology as described (Zhang et al., 2021). The chromatographic separation was conducted by using an Infinity Poroshell 120 pentafluorophenyl (PFP) column (2.1 × 100 mm, 1.9 µm) (Agilent, Santa Clara, CA, USA) and a binary mixture of water and acetonitrile, acidified with 0.1% (v/v) formic acid, as a mobile phase (LC-MS grade, VWR, Milan, Italy). The mass spectrometer was operated in full-scan mode (100–1200 m/z) and in positive polarity with a minimal resolution at 30,000 Full Width at Half Maximum (FWHM). The sequence was injected in a randomized way, and the Quality Control samples (QCs), made by pooling an aliquot of extract from each sample, were injected at the beginning of the sequence and every 6 sample injections. Raw metabolomics alignment was accomplished using Agilent’s Profinder B.07 software (Santa Clara, CA, USA). Annotation of the dataset was performed with the aid of the comprehensive PlantCyc 12.6 database from the Plant Metabolic Network (http://www.plantcyc.org), employing the ‘find-by-formula’ algorithm, which considers both monoisotopic accurate mass and the entire isotopic distribution, as described by Schläpfer et al. (2017). In our untargeted conditions, a level 2 of annotation (*i.e.*, putatively annotated compounds) was achieved, as reported by the COSMOS Metabolomics Standards Initiative (Salek et al., 2013). Compounds annotated in at least 75% of replicates within at least one treatment were retained for subsequent statistics.

### 2.5 Statistical analysis

Data analysis was performed considering at least four independent biological replicates for each treatment. The normality of data distribution and homogeneity of variances were assessed using the Shapiro-Wilk and Brown-Forsythe tests, respectively, by the software SPSS Statistics 26.0 (Version 26 SPSS Inc., Chicago, IL, USA), before evaluating the significance of the impact of the treatments (C, D, Zn, and D + Zn) on morphological, physiological, and biochemical parameters. Based on these results, parametric tests of one-way ANOVA followed by Tukey’s HSD (honestly significant difference) test were used to evaluate whether treatment types differ. Statistical significance was determined at *p* < 0.05. More details are provided in the **Supplementary File 1** and the caption text of relevant figures. Figures were generated using GraphPad Prism 8 (GraphPad Software, San Diego, CA, USA).

A Mass Profiler Professional B.12.06 from Agilent (Santa Clara, CA, USA) was used to perform chemometrics interpretation of metabolite profiles, as well as data transformation and normalization as described in earlier work (Ceccarelli et al., 2021). Based on fold-change data, an unsupervised hierarchical cluster analysis (HCA, Euclidean distance) was performed. After that, SIMCA 16 was used to perform supervised orthogonal projections to latent structures discriminant analysis (OPLS-DA), multivariate statistics analysis (Umetrics, Malmö, Sweden). Model fitness parameters were determined there (goodness of fit: R^2^Y; goodness of prediction: Q^2^Y; cross-validation: CV-ANOVA, *p* < 0.01). To validate and examine outliers, the permutation test (*n* = 200) and Hotelling’s T2 (95% and 99% confidence limits for the suspect and strong outliers, respectively) were used. Then, to find discriminant metabolites, the variable importance in the projection (VIP > 1.3) method was applied.

Lastly, differential compounds were identified through ANOVA and FC analysis criteria (*p*-value < 0.05 with Bonferroni multiple testing correction, fold-change > 2.5). Statistically significant compounds (*p*-value 0.05 and FC 2.5) were uploaded into PlantCyc’s Omic Viewer Pathway Tool (Stanford, CA, USA) to determine the pathways and processes impacted by treatments (Caspi et al., 2013).

## 3 Results

### 3.1 Stress effects on growth and root biomass

**Figure 1:**
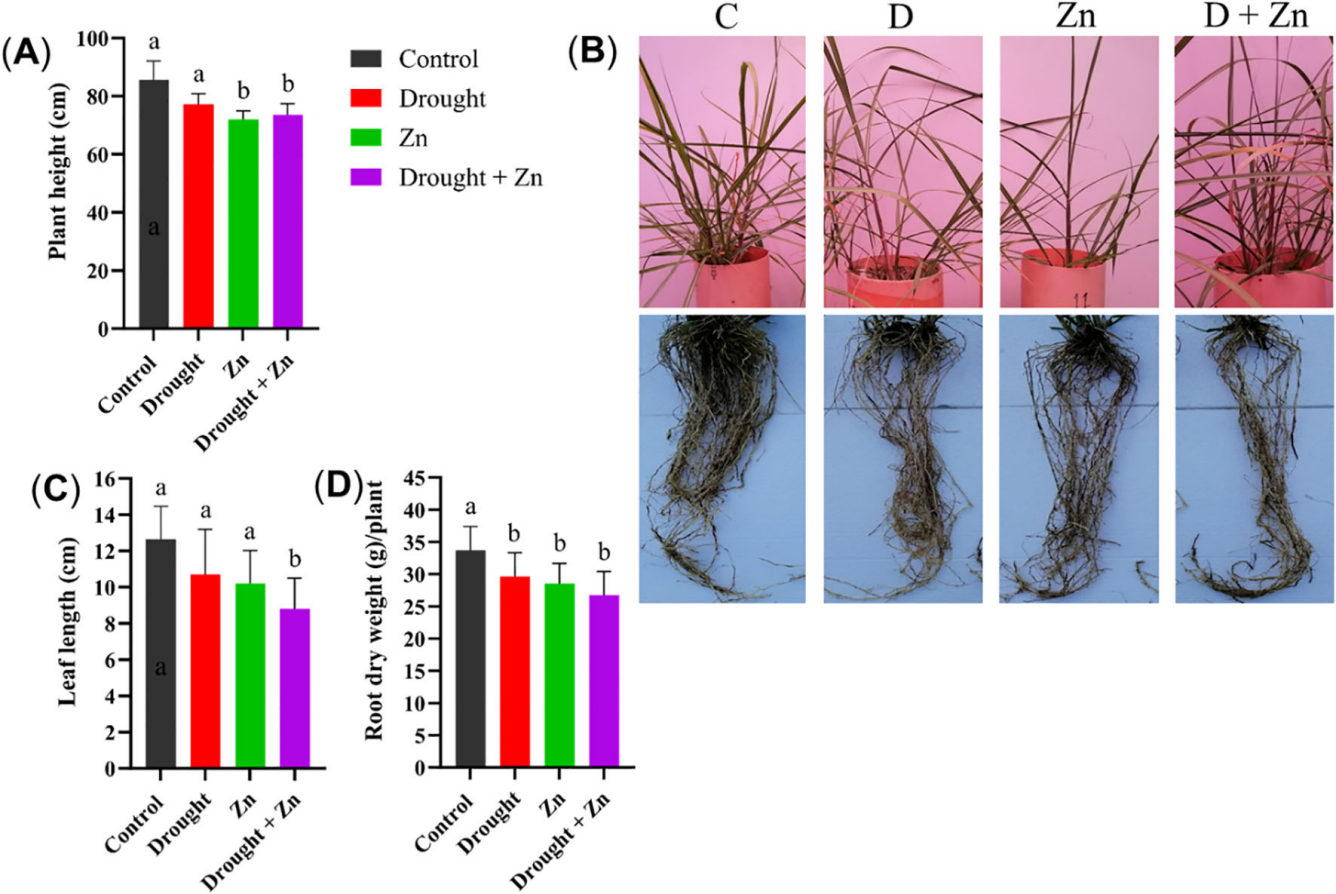
Morphological measurements of *M. sinensis* x *M. sacchariflorus* hybrid under Control **(C)**, drought **(D)**, zinc (Zn), and drought and zinc (D + Zn) conditions. In the panels, they show **(A)** plant height (cm), **(B)** images of the above-ground parts and roots after cleaning, **(C)** leaf length (cm), and **(D)** root dry weight in grams (g) per plant. Data are presented as mean ± SD (mean ± deviation, *n* = 4), and different letters **(a, b)** indicate a significant difference among the treatments according to Tukey’s HSD test at *P* < 0.05, one-way ANOVA: Plant height, F (3, 12) = 8.31, *p* = 0.0029, Leaf length, F (3, 12) = 4.39, *p* = 0.0264, Root dry weight, F (3, 12) = 3.83, *p* = 0.0390.

Stress treatments affected above and below-ground morphology under drought and Zn or combined stress (**Figure 1B**). Plant height was significantly reduced under Zn (*p* = 0.0031) and combined D + Zn stress (*p* = 0.0142), while D had less impact (*p* < 0.05), resulting in a moderate decrease in plant height compared to the control (**Figure 1A**). Together with representative images of plant morphology (**Figure 1B**), the leaf length was slightly reduced under drought and Zn stress (*p* < 0.05), but combined D + Zn stress significantly reduced leaf length (*p* = 0.0262) (**Figure 1C**). However, root dry biomass exhibited a decrease under all stress conditions (D, *p* = 0.048, Zn, *p* = 0.044, D + Zn, *p* = 0.041), in comparison to control plants (**Figure 1D**).

### 3.2 Physiological response to stress

**Figure 2:**
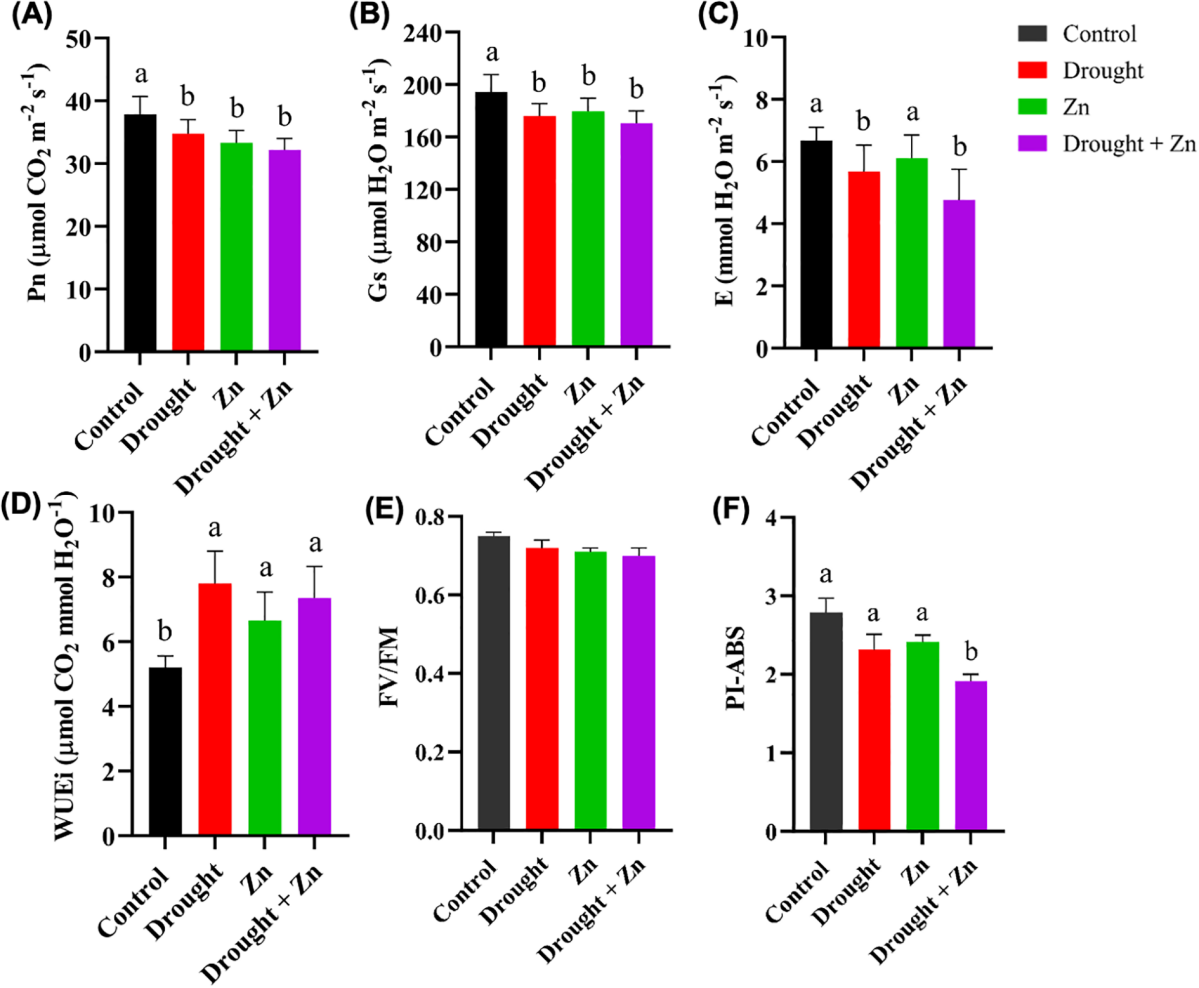
The gas exchange parameters: **(A)** Net photosynthesis rate (Pn, µmol CO_2_ m^-2^ s^-1^), **(B)** Stomatal conductivity (Gs, µmol H_2_O m^-2^ s^-1^), **(C)** transpiration rate (E, mmol H_2_O m^-2^ s^-1^) and **(D)** Intrinsic water use efficiency (WUEi, µmol CO_2_ mmol H_2_O^-1^); and chlorophyll fluorescence: **(E)** The maximum quantum efficiency of the PSII (Fv/Fm), **(F)** Performance index per absorbed light (PI-ABS) of *M. sinensis* x *M. sacchariflorus* hybrid under control, drought, Zn and drought + Zn stress conditions. Data are presented as mean ± SD (*n* = 4), and different letters **(a, b)** indicate a significant difference among the treatments according to Tukey’s HSD test at *P* < 0.05, one-way ANOVA: Pn, F (3, 12) = 5.33, *p* = 0.0145, Gs, F (3, 12) = 5.70, *p* = 0.0116, E, F (3, 12) = 5.05, *p* = 0.0172, WUEi, F (3, 12) = 6.4, *p* = 0.0078, PI-ABS, F (3, 12) = 5.28, *p* = 0.0148.

Photosynthetic leaf gas exchange is the major process for all metabolic processes in plants. After the drought, Zn and combined D + Zn stress, the net photosynthesis rate (Pn), stomatal conductance (Gs), transpiration rate (E), and intrinsic water use efficiency (WUEi) were determined. Significant differences (*p*-value < 0.05) in leaf gas exchange parameters were observed under D, Zn, and combined D + Zn stress (**Figures 2A–D**). The net photosynthesis rate (Pn), stomatal conductance (Gs), and transpiration rate (E) exhibited significant variations under D, Zn, and combined D + Zn stress conditions, compared to the control. Pn significantly reduced under D (*p* = 0.0452), Zn (*p* = 0.0439), and combined D + Zn (*p* = 0.0158), Gs significantly reduced under D (*p* = 0.0282), Zn (*p* = 0.0416) and combined D + Zn (*p* = 0.0305), and E significantly reduced under D (*p* = 0.0465) and D + Zn (*p* = 0.0206) compared to the control (**Figures 2A–C**). In contrast, no significant difference was observed in transpiration rate under Zn stress (*p* < 0.05), compared to the control (**Figure 2C**). However, plant-induced iWUE in all stress conditions, specifically D (*p* = 0.0491), Zn (*p* = 0.0432), and combined D + Zn stress (*p* = 0.0073) conditions, compared to the control (**Figure 2D**). This suggests that *M. sacchariflorus* × *M. sinensis* hybrid enhances water-use efficiency as a part of an adaptive response to limited water availability and stomatal regulations.

Chlorophyll fluorescence parameters, including Fv/Fm and PI-ABS, showed no significant differences (*p* < 0.05) in stress-treated plants when compared to the control group (**Figures 2E-F**). Chlorophyll fluorescence levels remained consistent at 0.75 Fv/Fm across all leaves under controlled conditions. A marginal decrease to Fv/Fm 0.74 to 0.73 was observed under drought, zinc, and combined stress conditions, albeit not reaching statistical significance (**Figure 2E**). The calculated Performance index per absorbed light (PI-ABS) was slightly more sensitive to combined D + Zn treatment (*p* = 0.0155) (**Figure 2F**). The *Miscanthus*, *M. sacchariflorus* × *M. sinensis* hybrid sustained photosynthesis longer under severe stress but was somewhat sensitive to combined D + Zn stress. This resilience was supported by enhanced induction of protective mechanisms like non-photochemical quenching.

### 3.3 Biochemical responses to recurrent stress

#### 3.3.1 Effect of drought and zinc stress on MDA and proline content

**Figure 3:**
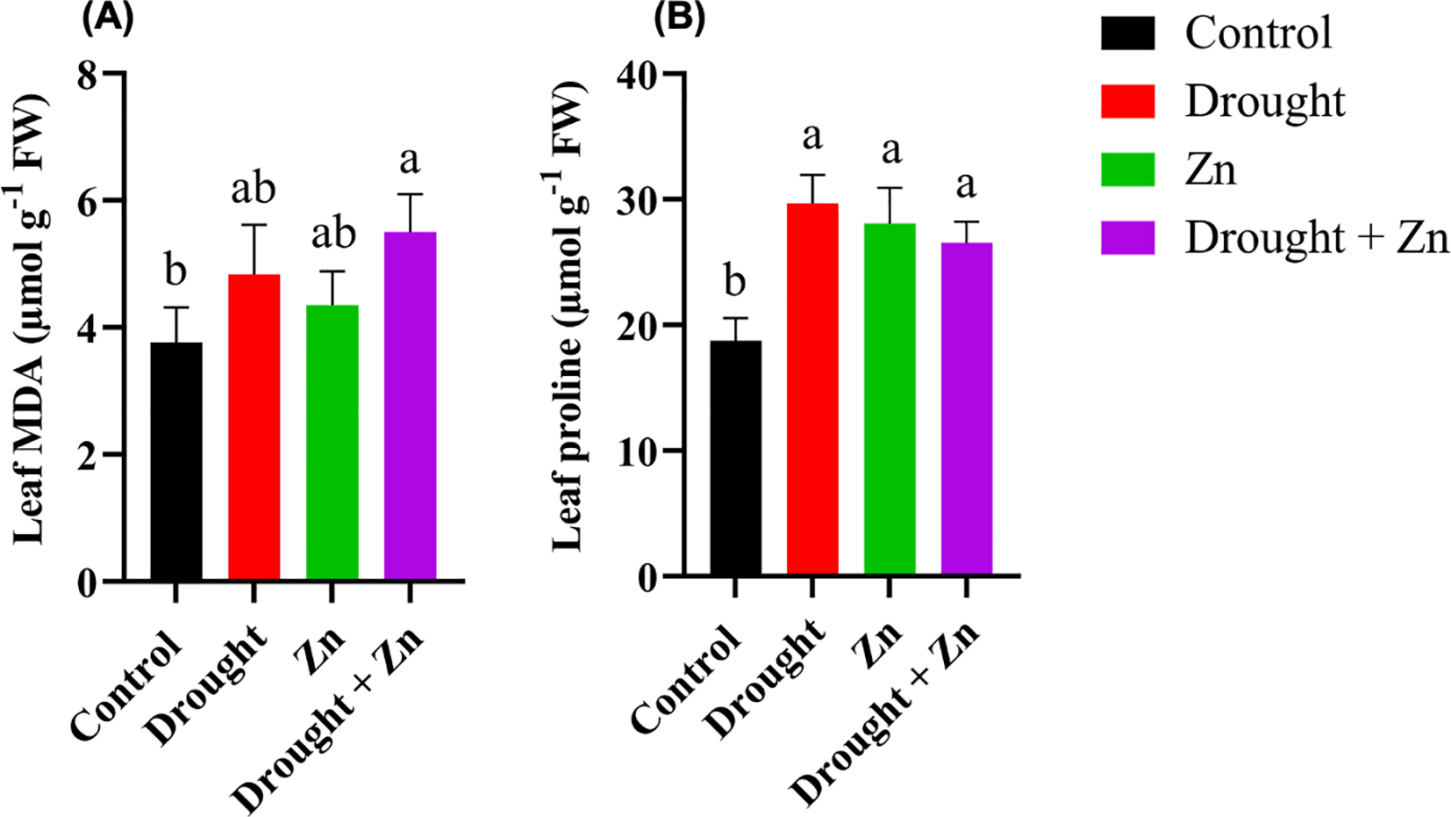
Effect of D, Zn, and combined D + Zn on malondialdehyde (MDA) **(A)** and proline contents **(B)** in the leaf of *M. sinensis* x *M. sacchariflorus* hybrid under terminal all stress and control conditions. Data are presented as mean ± SD (*n* = 4), and different letters **(a, b)** indicate a significant difference among the treatments according to Tukey’s HSD test at *P* < 0.05, one-way ANOVA: MDA, F (3, 12) = 5.40, *p* = 0.0139, Proline, F (3, 12) = 19.64, *p* < 0.0001.

Biochemical profiling of *M. sacchariflorus* × *M. sinensis* leaves following stress treatments revealed distinctions between control and treated plants regarding their ability to trigger protective mechanisms. MDA, an oxidation byproduct of unsaturated lipids, accumulates in response to oxidative stress (Kang et al., 2020). MDA content exhibited a significant increase under the combination of D + Zn stress treatment (*p* = 0.0146) compared to control plants, while a slight increase occurred under drought and zinc treatments (**Figure 3A**). Proline is a well-established compatible osmolyte that accumulates in plants in response to a range of abiotic stresses, mostly with drought, Zn, and salt stress (Zhou et al., 2014; Islam et al., 2015). Proline was also increased in leaves under all stress conditions (*p* < 0.05), and this trend of increasing proline followed that of MDA. Among D (*p* < 0.0001), Zn (*p* = 0.0004), and combined D + Zn (*p* = 0.0017) treatments, caused a dramatic increase (up to 58%) in proline accumulation when compared to control plants (**Figure 3B**).

#### 3.3.2 Activity of antioxidant enzymes

**Figure 4:**
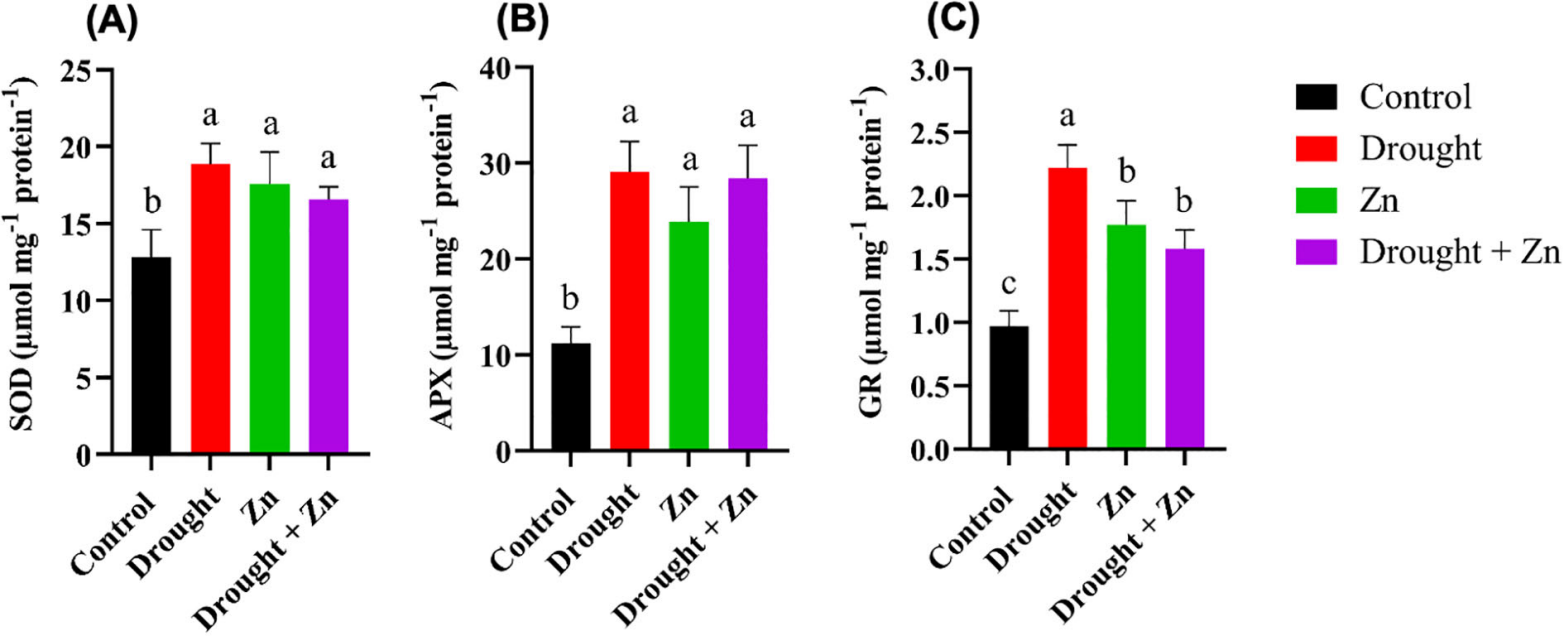
Response of **(A)** Superoxide dismutase (SOD) (µm proteins); **(B)** Ascorbate peroxidase (APX); **(C)** Glutathione reductase (GR) enzymes in leaves of *M. sinensis* x *M. sacchariflorus* hybrid to control, D, Zn, and D + Zn stress conditions. Data are expressed with mean ± SD (*n* = 4), and different letters **(a, b, c)** indicate a significant difference among the treatments according to Tukey’s HSD test at *P* < 0.05, one-way ANOVA: SOD, F (3, 12) = 10.98, *p* = 0.0009, APX, F (3, 12) = 28.65, *p* < 0.0001, GR, F (3, 12) = 40.18, *p* < 0.0001.

To investigate whether plants adjust their antioxidant metabolism in response to potential increases in oxidative stress, we conducted assays to quantify the activities of pivotal antioxidant enzymes, including superoxide dismutase (SOD), ascorbate peroxidase (APX), and glutathione reductase (GR). In general, the activity of SOD, APX, and GR increased under D, Zn, and combined D + Zn stress conditions (**Figures 4A–C**). SOD activity was increased 48%, 37%, and 30% under D (*p* = 0.0009), Zn (*p* = 0.0066), and combined D + Zn stress (*p* = 0.0311), respectively, compared to the control plants (**Figure 4A**). Similarly, APX and GR activities increased significantly after drought stress. Particularly, APX activity exhibited a notable increase (up to 159%) under drought stress (*p* < 0.0001), Zn (*p* = 0.0005, up to 113%), and up to 153% in the presence of D + Zn (*p* < 0.0001) (**Figure 4B**). In contrast, the maximum GR activity was detected under drought stress (*p* < 0.0001, up to 128%) and Zn stress (*p* < 0.0001, up to 82%), and combined D + Zn (*p* = 0.0012, up to 62%) compared to control plants (**Figure 4C**).

### 3.4 Metabolite profile recurrent to drought and Zn stress

**Figure 5:**
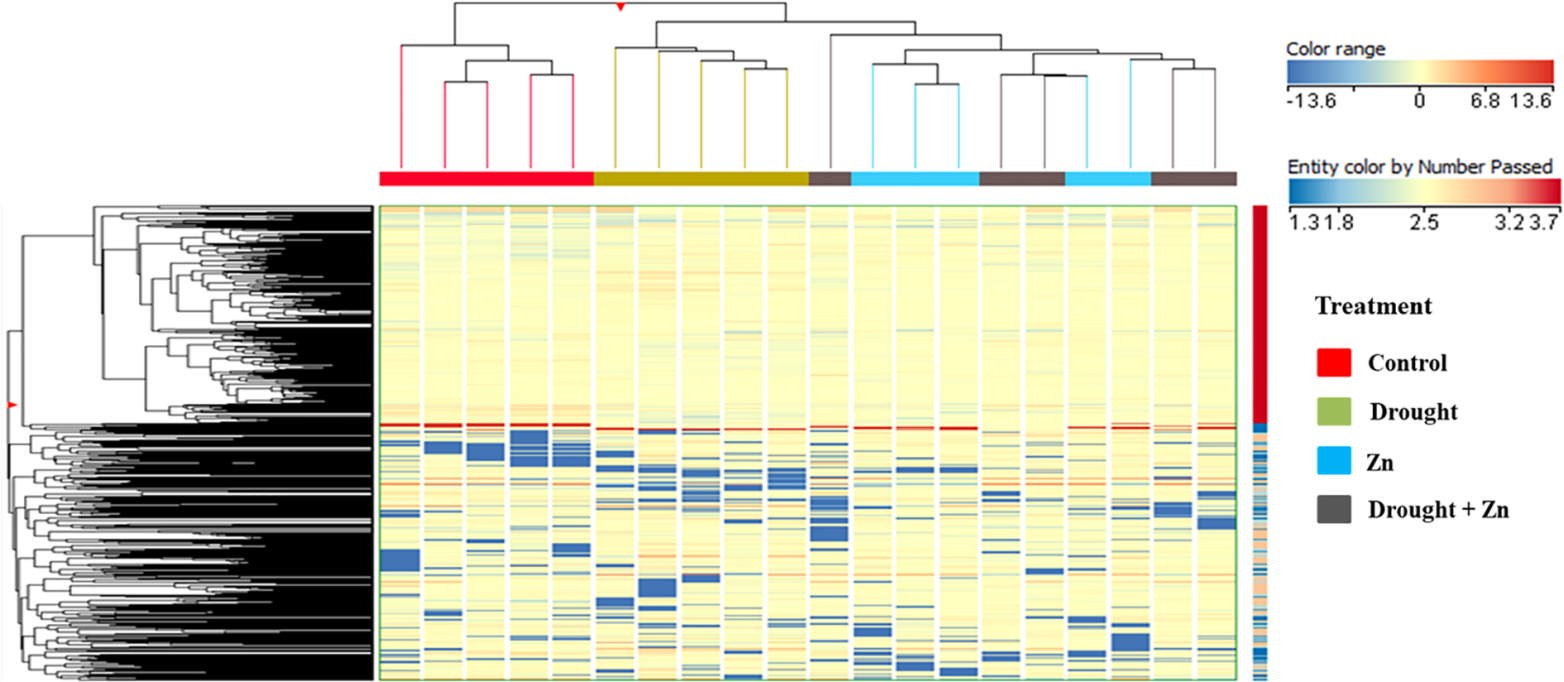
Chemometric results on the untargeted metabolomics profile of *M. sinensis* x *M. sacchariflorus* leaves under D, Zn, and combined D + Zn stress compared to control plants. Unsupervised hierarchical cluster analysis (HCA) based on fold change heatmap (Euclidean distance, Ward’s linkage rule) was carried out from UHPLC-ESI/QTOF-MS metabolomics analysis.

Untargeted metabolomics using UHPLC-QTOF-MS was conducted to shed light on the further biochemical processes involved in mitigating the D, Zn, and combined D + Zn stress in the *M. sacchariflorus* × *M. sinensis* hybrid. Untargeted metabolites profiling allowed us to annotate a total of 2,667 compounds, and the list of annotations is presented in the **Supplementary Material**, accompanied by individual abundance data, retention time, and composite mass spectra (**Supplementary Table S1** in Supplementary file 2). As an initial pattern exploration, unsupervised hierarchical cluster analysis (HCA) was conducted to elucidate metabolomic patterns and discern similarities and differences among treatments (**Figure 5**). The HCA produced two main clusters; the first cluster included control plants, while the second cluster was, in turn, composed of two different sub-clusters with D and Zn and combined D + Zn stress. The clustering results suggest that the *M. sacchariflorus* × *M. sinensis* hybrid shares a common metabolic response to D and Zn stress and combined D + Zn stress conditions, but these are distinct from control conditions. However, upon closer examination, each treatment formed a distinct cluster, thereby demonstrating hierarchically non-prevalent yet discernible metabolic profiles (**Figure 5**).

**Figure 6:**
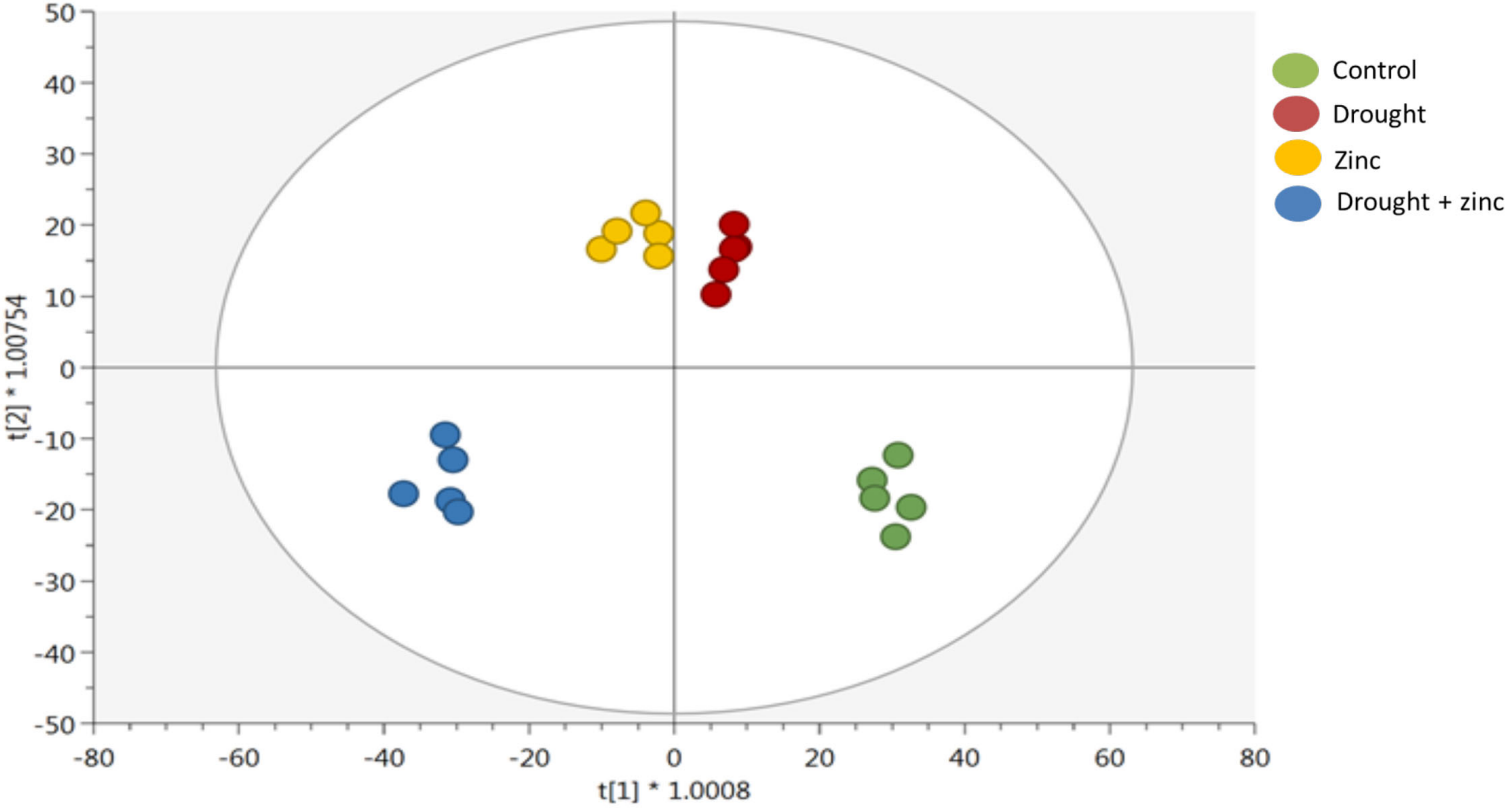
Orthogonal projection to latent structures discriminant analysis (OPLS-DA) supervised modelling according to first and second latent vectors, t[1] and t[2], respectively, of *M. sinensis* x *M. sacchariflorus* hybrid under control, D, Zn, and combined treatment (D + Zn). The model correlation with the dataset was R^2^Y = 0.99, whereas the prediction ability was Q^2^Y = 0.93 (*n* = 5).

Supervised OPLS-DA modelling was then implemented to identify discriminant metabolites that were significantly contributing to the distinctions among the groups in a supervised manner (**Figure 6**). The goodness of prediction and goodness of fit parameters of the model were Q^2^Y = 0.702 and R^2^Y = 0.976, respectively. In addition, OPLS was confirmed by HCA by obtaining groups of different clusters. The t[1] component result confirmed a distinctive metabolomic profile of *Miscanthus* treated with stress combination (D + Zn), while the t[2] component showed that Zn and drought-treated plants showed distinctive metabolomics profiles compared to control plants. Stress-treated plants displayed different metabolomics profiles compared to control plants. The list of discriminative metabolites, along with their VIP^2^ scores (by setting the VIP score threshold to >1.3), is presented in **Supplementary Table S3** (Supplementary file 2). These VIP compounds included those involved in fatty acid and lipid biosynthesis, as well as secondary metabolism, including multiple cofactors. The most distinctive VIP compounds found in secondary metabolites were diterpenoids, triterpenoids, alkaloids, small molecules, anthocyanin, soyasaponin, and coenzyme A-activated compounds.

**Figure 7:**
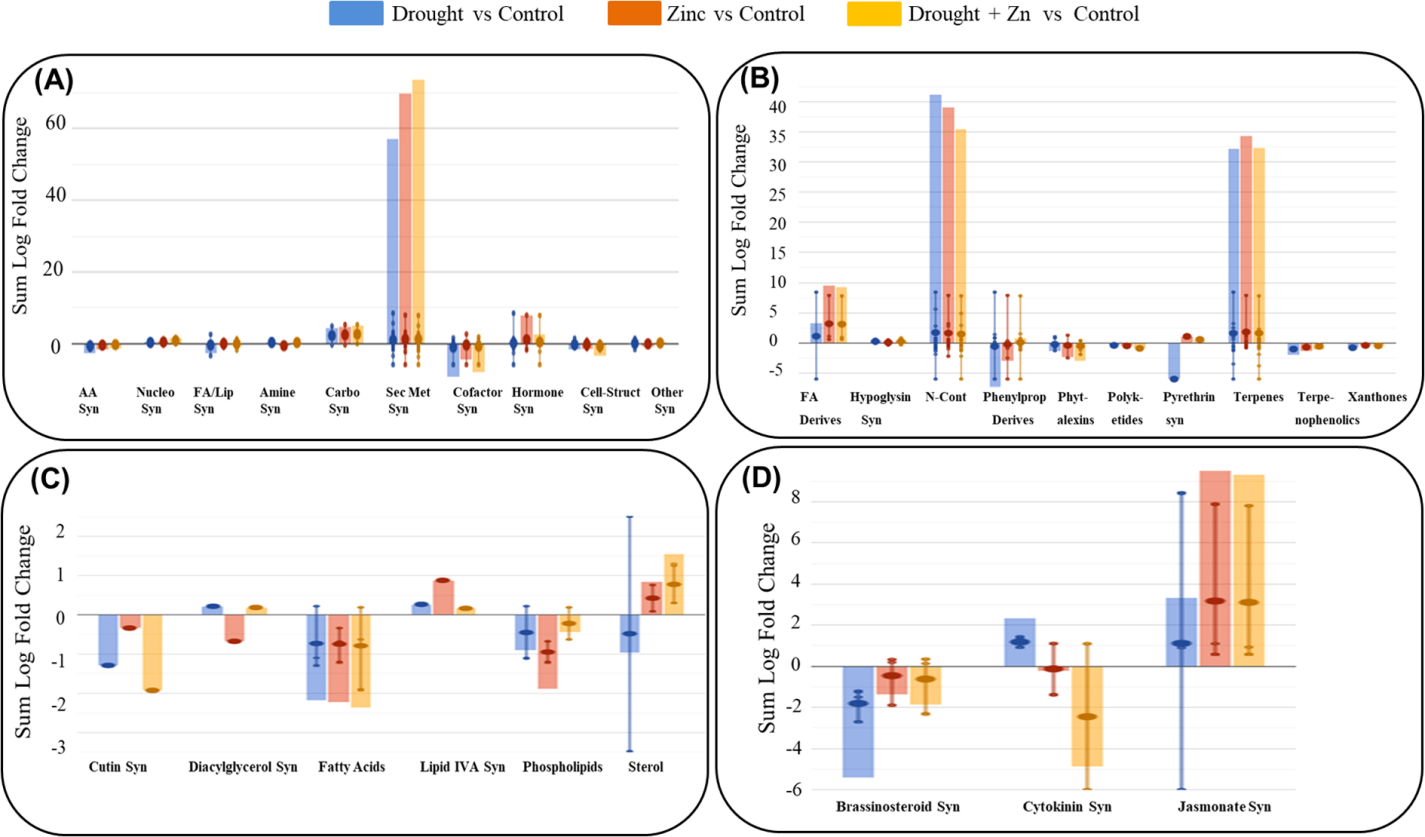
Pathway analysis of *M. sinensis* x *M. sacchariflorus* hybrid leaves under D, Zn, and combined D + Zn stress conditions compared to the control plant leaves. The metabolites that passed the fold change (FC ≥ 2.5) and ANOVA (*p*-value < 0.05) analyses were employed to perform the pathway analysis. Differential metabolites were interpreted in terms of biosynthesis pathways **(A)**, Secondary metabolites **(B)**, Fatty acid and Lipid biosynthesis **(C)**, and Hormone biosynthesis pathway **(D)**. The error bars depict the standard deviation of logFC (log-fold change) values across all compounds within the respective class. The larger dot represents the median value, while the smaller dot represents the actual value of each compound in the class. The abbreviated subcategory names on the x-axis correspond to: AA: amino acids synthesis; Nucleo: nucleosides and nucleotides; FA/Lipids: fatty acids and lipids; Amines: amines and polyamines; Carbo: carbohydrates; Cofactors: cofactors, prosthetic groups, electron carriers, and vitamins; Cell-Struct: Cell structures; FA: fatty acids; N- containing: nitrogen-containing; Syn: Synthesis.

Subsequently, a Volcano Plot analysis was employed to explore the underlying mechanism of D, Zn, and combined D + Zn stress on *M. sacchariflorus* × *M. sinensis* hybrid, integrating ANOVA statistical analysis (*p* < 0.05) and fold change analysis (with a threshold of 2.5). In total, 139 metabolites exhibited differential expression were identified, and their comprehensive list is available in the Supplementary Material (**Supplementary Table S2** in Supplementary file 2). The resulting biosynthetic pathways are represented graphically in **Figure 7**, which includes the biosynthetic pathways (**Figure 7A**), secondary metabolites (**Figure 7B**), lipids and fatty acids (**Figure 7C**), and hormones (**Figure 7D**). Additionally, a comprehensive summary pathway detailing the stress response is provided in **Table 1**, and in the supplementary file 2, **Supplementary Table S3**. In general, stress treatment elicited a significant enhancement of secondary metabolism in the *M. sacchariflorus* × *M. sinensis* hybrid, with 64 notable compounds implicated in this process. Greater modulations were observed in the accumulation of secondary metabolites, followed by hormones and carbohydrates under both D, Zn, and combined D + Zn stress compared to the control, as shown in **Figure 7A**. However, the synthesis of secondary metabolites exhibited a greater increase by 73.92 cumulative LogFC values under combined D + Zn stress compared to either D or Zn stress treatments alone. In the case of secondary metabolites, fatty acid derivatives, terpenes, and nitrogen-containing secondary metabolites accumulated highly under D, Zn, and combined D + Zn stress treatments (**Figure 7B**). Meanwhile, N-containing metabolites were more abundant under drought as compared to Zn and in combined D + Zn stress. Stress treatment also modulated fatty acid and lipid biosynthesis pathway, as sterol showed up-accumulation in Zn and combined stress plants, as well as lipid IVA synthesis, also showed up-accumulation under drought, Zn and D + Zn stress conditions (**Figure 7C**). However, cutin, diglycerol, and phospholipid synthesis were reduced in abundance under all stress conditions. The biosynthetic pathways of phytohormones were influenced by treatment with D, Zn, and combined D + Zn stress conditions compared to control plants. In particular, cytokinin showed increased abundance in response to drought stress. Meanwhile, jasmonate also showed increased accumulation under all stress conditions and especially in Zn and in combined D + Zn stress conditions (logFC = 9.8 and 9.3, respectively) (**Figure 7D**). However, other hormones, such as brassinosteroid, showed reduced relative abundance under stress conditions, and cytokinin showed decreased abundance under Zn and combined D + Zn stress conditions compared to control plants.

**Table 1:** Summary of different pathways activated in *M. sinensis* x *M. sacchariflorus* hybrid under D, Zn, and combined D + Zn stress conditions.

Name of compounds	Drought *vs.* control			Zn *vs.* control			Drought + Zn *vs.* control		
	No of compounds	Average LogFC	Sum LogFC	No of compounds	Average LogFC	Sum LogFC	No of compounds	Average LogFC	Sum LogFC
Amino acid	4	-0.72	-2.89	4	-0.52	-2.08	4	-0.36	-1.47
Nucleosides and nucleotides	2	0.19	0.39	2	0.35	0.71	2	0.74	1.48
Fatty acid and Lipid	6	-0.48	-2.89	6	-0.08	-0.51	6	-0.1	-0.65
Amines and polyamines	1	0.21	0.21	1	-0.67	-0.67	1	0.18	0.18
Carbohydrates	2	2.16	4.33	2	2.39	4.78	2	2.53	5.07
Secondary metabolites	64	0.89	57.25	64	1.11	70	64	1.15	73.92
Cofactors, Electron carriers	10	-1.07	-10.73	10	-0.46	-4.6	10	-0.81	-8.16
Hormones	8	0.03	0.29	8	0.98	7.89	8	0.32	2.56
Cell structures	5	-0.35	-1.75	5	-0.31	-1.58	5	-0.74	-3.71
Other biosynthesis	5	-0.01	-0.09	5	-0.18	-0.93	5	0.06	0.33

For each pathway highlighted under the different treatments, the number of compounds involved, the average Log fold-change value (LogFC), and the sum LogFC are provided. The LogFC values refer to a pairwise comparison between each treatment *vs.* the control, non-treated.

## 4 Discussion

### 4.1 Adaptive strategies to support growth and survivability under drought and heavy metal stress

Plant growth, development, survival, and crop production can be adversely affected by drought stress and the accumulation of heavy metals (HMs) in the soil, which pose a substantial threat to sustainable agriculture. Considering the devastating effects of climate change, abiotic resilience is becoming increasingly important and desirable. However, Zn is universal in the environment, comprising up to 0.02% of the Earth’s crust by weight, and global topsoil averages 75 mg kg^-^¹, whereas European agricultural topsoil is typically 45–50 mg kg^-^¹ (Rodrigues et al., 2010). Zn concentrations can vary due to differences in parental material or anthropogenic input. At the local scale, Europe has comparatively greater Zn concentrations because of the existence of Zn-rich geological formations, such as those in Portugal, Spain, Sardinia, Ireland, and Poland (Van Eynde et al., 2023). The applied Zn concentration (400 mg kg^-^¹) is in the severely contaminated range for agricultural soils, thereby creating a strong stress context to resolve tolerance responses in the *Miscanthus* hybrid. Plants have evolved a variety of strategies depending on several variables, including species, developmental stage, and duration of stress (Stavridou et al., 2019; Qin et al., 2022). Drought and Zn stress lead to decreased plant biomass due to the increased energy expenditure in dealing with oxidative stress rather than biomass production. Different plant species and hybrids cope with this stress in different ways. In plants, the regulation of leaf water potential by stomata can be described as a spectrum ranging from isohydric to anisohydric plant behavior (Salvi et al., 2022). Isohydric plants aim to maintain consistent midday leaf water potential (Ψ_leaf_) under both well-watered and drought conditions by adjusting stomatal conductance to minimize transpiration, whereas anisohydric plants keep stomata open for extended periods to maintain high photosynthetic rates even as leaf water potential decreases (Chaves et al., 2016). However, other characteristics, such as anatomical and morphological features, stomata density and morphology, and gas exchange measurements *in vivo*, different secondary metabolites also help place plants within a continuum of stress responses (Ings et al., 2013; Zhang et al., 2021).

### 4.2 Stress-specific and combined effects on plant growth, morphology and biomass

A set of recently developed *Miscanthus* hybrids has been evaluated and ranked for drought and Zn stress tolerance based on their morpho-physiological and biochemical responses (Islam et al., 2023). In the current study, we investigated the ecophysiological, biochemical, and metabolic profiles of the most drought and Zn stress-tolerant *M. sacchariflorus* × *M. sinensis* GRC10 hybrid to unravel their stress tolerance mechanisms. The initial physiological response observed in the GRC10 hybrid under drought, Zn stress, and their combination (D + Zn) included a reduction in leaf length, accompanied by a moderate decrease in plant height. In contrast to plants subjected to Zn stress alone and the combined D + Zn stress, those treated solely with drought exhibited a lesser impact on stem elongation, plant height, and, consequently, root dry weight biomass. This observation underscores the inherent resistance of *Miscanthus* to drought stress. *Miscanthus* is grown specifically for its lignocellulosic biomass, with the entirety of its above-ground biomass harvested (Robson et al., 2013). As a result, we anticipated notable correlations, especially between the rate of stem elongation by plant height under stress conditions and the overall yield in the *M. sacchariflorus* × *M. sinensis* hybrid.

### 4.3 Photosynthetic performance, gas exchange, and improved iWUE under individual drought, Zn and combination stress in *M. sacchariflorus* × *M. sinensis*

Tolerance to diverse environmental conditions depends on the management of leaf gas exchange parameters such as Pn, Gs, E, and WUEi (Hamani et al., 2021). According to our analysis of the experimental treatments, drought stress, Zn, and combined D + Zn stress significantly affected gas exchange parameters in *Miscanthus M. sacchariflorus* × *M. sinensis* leaves (**Figure 2**). A similar observation was found in different *Miscanthus* hybrids (conventional *Miscanthus* × *giganteus*) under drought and trace element metal stress (Krzyżak et al., 2023). A critical strategy for surviving drought is to reduce stomatal conductance, which is the primary mechanism for avoiding dehydration (Reynolds-Henne et al., 2010). Here, *M. sacchariflorus* × *M. sinensis* hybrid reduced stomatal conductance (Gs) in the D, Zn, and combined D + Zn stress, leading to a pronounced reduction in the transpiration (E) and photosynthesis rate (Pn) in the leaf, consistent with previous findings that tolerant plants utilize stomatal closure as a strategy to conserve water and maintain photosynthesis, thus enhancing their survival under limited water conditions (Stavridou et al., 2019; El-Esawi et al., 2020). On the other hand, iWUE increased under all stress conditions, especially under drought and combined D + Zn stress compared to control plants, which could be attributed to maintaining high tissue water content in limited water availability.

Chlorophyll fluorescence stands out as one of the select physiological parameters known to correlate significantly with abiotic stress tolerance (Belkhodja et al., 1994). Assessing the integrity of the photosynthetic apparatus, PSII maximum quantum efficiency (F*_v_/*F*_m_*) of photosynthetic performance was not affected by D, Zn, or combined D + Zn stress (**Figure 2E**). A similar observation was found under salinity and drought stress on *M. sinensis* (Sun et al., 2016; Stavridou et al., 2019), suggesting a tolerance mechanism of the *M. sacchariflorus* × *M. sinensis* hybrid to maintain the photosynthetic efficiency. However, PI was found to exhibit slightly more sensitivity than the maximum quantum yield of PSII (*F*_v_/*F*_m_). The PI decreased moderately under drought and Zn stress (not significantly, *p* > 0.05), significantly under combined D + Zn stress (*p* = 0.0155). The PI-ABS parameter provides more comprehensive, encompassing absorption and trapping of excitation energy, electron transport beyond the primary plastoquinone, and excitation energy dissipation (Oukarroum et al., 2007).

### 4.4 Oxidative stress responses and antioxidant enzyme activity

When plants are exposed to drought and HMs, including Zn stress, they produce MDA because of ROS-induced lipid peroxidation, which can be used as a stress indicator to evaluate plasma membrane damage and plant stress tolerance (Mengqi et al., 2016). To adapt to drought and Zn stress, plants activate the induction of antioxidant enzymatic machinery to cope with oxidative stress. Proline is a crucial osmolyte that plays an important role in osmotic adjustment, scavenging of ROS, stabilization of proteins and cellular structures, and protecting the photosynthetic apparatus under adverse conditions (Hayat et al., 2007; Stavridou et al., 2019). In our study, proline accumulation was observed in the Bates assay under single drought, Zn, and D + Zn combined stress in *M. sacchariflorus* × *M. sinensis* hybrid GRC10, which might modulate a decrease in MDA content under such stresses, potentially protecting the membranes from lipid peroxidation (**Figure 3A**, 3B). The Bates assay measures total ninhydrin-reactive compounds (including proline derivatives), whereas UHPLC-QTOF-MS specifically measures free L-proline. Therefore, the biochemical increase and metabolomic decrease are not contradictory but indicate distinct aspects of amino acid metabolism under stress. The accumulation of proline and decrease in MDA levels in tissues were also observed in *M. × giganteus* due to drought and Cd stress (Ings et al., 2013), similar to other drought-resistant varieties such as wheat (Islam et al., 2015), potato (Demirel et al., 2020), and tomato (Ceccarelli et al., 2021). The ability to accumulate proline varies among species and might play a role in stress tolerance, but it is not always necessary for adaptation to severe environmental conditions (Szabados et al., 2011).

Antioxidant enzymes play an important role in plant stress tolerance by mitigating the negative effects of ROS produced during stress conditions. The elevation of antioxidant enzymes, including SOD, POD, GR, and APX, has been reported in many drought-tolerant plant species, such as potato (Demirel et al., 2020), tomato (Mittova et al., 2002), under Cd, Pb, and Zn stress in *Miscanthus × giganteus* hybrid (Krzyżak et al., 2023). Antioxidants likely play a role in reducing stress-induced oxidative damage in plant cells. Our results are consistent with these findings, which showed that the antioxidant system in *M. sacchariflorus* × *M. sinensis* was increased in response to D, Zn, and combined D + Zn stress. In contrast, antioxidant enzymes may exhibit a notable reduction in activity under extreme abiotic stress conditions (Del Buono et al., 2021). We found that the activities of SOD, APX, and GR significantly rose in *M. sacchariflorus* × *M. sinensis* when exposed to D, Zn, and a combination of both stresses. Particularly, the enzymes were greatly stimulated by drought stress. In the presence of D, Zn, and combined D + Zn stress, the observed induction of APX, SOD, and GR activities, which most likely preserved the redox status within chloroplasts to improve photosynthetic rate and lead to increased chlorophyll content.

### 4.5 Coordinated hormonal and metabolic adjustments in response to drought, Zn, and combination (D + Zn) stress in *M. sacchariflorus* × *M. sinensis*

The occurrence of oxidative stress in plants can trigger metabolic reprogramming. ROS disrupts regular cellular functions and initiates alterations in metabolic pathways, affecting energy generation, nutrient utilization, and the synthesis of secondary metabolites. Different stresses activate different gene expressions and modify various metabolites such as amino acids, organic acids, and carbohydrates, which play key roles in various plant processes such as photorespiration and protein synthesis (Sairam et al., 2000; Merewitz et al., 2012). The biosynthesis pathway of secondary metabolites and metabolite profiles were induced under D, Zn, and combined D + Zn stress conditions. The most notable secondary metabolites found among the 64 identified were alkaloids, phenylpropanoids, terpenoids, as well as other compounds associated with chlorophyll, phytohormones, and polyamine biosynthesis. Specifically, plants highly accumulated nitrogen-containing secondary metabolites and terpenes (**Figure 7B**). Tolerance to a wide variety of stimuli is improved by the accumulation of flavonoids, terpenoids, and phenolic compounds. Fatemi et al. (2020) demonstrated that the glucosinolate glucomalcommin and its intermediates played a role in relieving drought stress and maintain plant growth through the regulation of water balance in plants. Thus, the accumulation of glucosinolate intermediates (3-(methylsulfanyl) propyl-desulfoglucosinolate, (methylsulfanyl) pentyl-thiohydroximate) might be a response to low water potential under D and combined D + Zn stress, which can lead to the synthesis of osmotically active molecules. Similarly, under abiotic stress, the phenylpropanoid biosynthetic pathway was activated, leading to the accumulation of phenolic substances with high antioxidant properties (Yadav et al., 2021). Additionally, the production of lipids, specifically through the creation of cutin synthase compounds, promotes the high levels of stomatal transpiration, according to Kerstiens (1997), Kerstiens, 2006). Drought stress enhances terpenoid biosynthesis (Ma et al., 2014). Drought was observed to cause transcriptional alterations associated with the synthesis of terpenes and flavonoids in multiple crops (Zhang et al., 2021; Zhang et al., 2023). Specifically, in Arabidopsis, astaxanthin (3S,3’S) was identified as playing a crucial role in mitigating drought-induced oxidative stress through its ability to enhance antioxidant capacity, reduce reactive oxygen species (ROS) production, and stabilize cell membranes (Yu et al., 2021). Under drought, Zn, and combination stress, *M. sacchariflorus* × *M. sinensis* induced a higher accumulation of astaxanthin (3S,3’S) and increased antioxidant activity, indicating a coordinated photoprotective response against oxidative stress.

Cytokinin (CK) and jasmonate (JA) play a critical role in drought stress tolerance in plants by regulating various physiological and molecular processes, including stomatal regulation, root growth, osmotic stress responses, and antioxidant defense mechanisms. JA is also known to increase different secondary metabolites, such as amino acids and alkaloids (Wasternack and Hause, 2013). Several studies showed that JA can diminish the adverse impact of drought on the rate of photosynthesis, highlighting its potential as a facilitator of plant resilience under drought stress conditions (Yoon et al., 2009; Javid et al., 2011). Higher accumulation of jasmonate-3-oxo-2-(cis-2’-pentenyl)-cyclopentane-1-(3-oxobutanoyl)-CoA in D, Zn, and combined D + Zn might potentially serve as signaling molecules to activate ROS scavenging antioxidant enzymes, such as SOD, APX, and GR activities, as well as decreased MDA contents. However, both drought and Zn stress, as well as their combined D + Zn stress, result in a general increase in the pool of amino acids and hormone/signaling-related metabolites, which is due to the well-known influence of abiotic stress to stimulate fundamental metabolic processes of stress tolerance in *M. sacchariflorus* × *M. sinensis*.

### 4.6 Implications for bioenergy crop improvement and stress resilience

The GRACE field trials, with published data for 2018-2021 (Awty-Carroll et al., 2023), showed that *M. sacchariflorus* × *M. sinensis* hybrid GRC10 seedlings raised as plug plants in the glasshouse established faster and were more tolerant to drought stress than the standard clone, *Miscanthus* × *giganteus* (GRC9), planted by rhizomes. *M. sacchariflorus* × *M. sinensis* performed particularly well in the trial in Northern Italy. In this paper, the potential for extending the growing areas of *M. sacchariflorus* × *M. sinensis* on Zn-contaminated and drought-prone sites was explored by performing a pot experiment in a controlled environment to reduce the complexity of environmental factors associated with field trials. This revealed a plethora of resilience mechanisms. A next step would be to grow *M. sacchariflorus* × *M. sinensis* on several contaminated and drought-prone field sites in Italy to test if these advantages translate to the field. If consistent positive field results are obtained, then this will be highly encouraging and informative to the breeders who could use these results to make further screens, selections, and crosses to further improve the supply of hybrids better adapted for growth on heavy metal-contaminated and marginal lands.

## 5 Conclusion

Metabolomics in *Miscanthus* hybrids not only serves the drought and Zn stress tolerance mechanisms but also identifies biochemical markers for maximizing biomass yield ‘in and on’ drought-prone marginal lands. This study delves into the intricate interplay of physiological, biochemical, and metabolomic responses in a *M. sacchariflorus* × *M. sinensis* hybrid under D, Zn, and combined stress conditions (D + Zn). Our findings indicate that the *M. sacchariflorus* × *M. sinensis* GRC10 hybrid can maintain higher photosynthetic performance of quantum yield of PSII, reduced transpiration rates through stomatal regulation, increased antioxidant defense mechanisms, mitigating oxidative stress, and thereby maintain biomass under D, Zn, and combined D + Zn stress. The metabolic reprogramming, assessed via HCA and OPLS-DA, revealed distinct effects of these different stresses. Prominently altered metabolites included those involved in carbohydrate biosynthesis, osmolyte production, and secondary metabolism pathways like glucosinolates and phytohormones in response to both D and Zn stress, suggesting a common interplay of effects associated with D and Zn stress tolerance in the *M. sacchariflorus* × *M. sinensis* GRC10 hybrid. Further investigations into the underlying molecular mechanisms and their implications for plant biomass production and stress resilience are needed to advance our efforts to breed hybrids with improved drought and heavy metal adaptation to allow sustainable biomass production on marginal lands and harsh environments.

## Data Availability

The original contributions presented in the study are included in the article/**Supplementary Material**. Further inquiries can be directed to the corresponding author.
